# RISK FACTORS AND IMPACT OF FINANCIAL FRAUD ON OLDER ADULTS AND DISADVANTAGED COMMUNITIES

**DOI:** 10.1093/geroni/igac059.1600

**Published:** 2022-12-20

**Authors:** Marguerite DeLiema, Paul Witt

**Affiliations:** University of Minnesota, Twin Cities, Minneapolis, Minnesota, United States; Federal Trade Commission, Washington, District of Columbia, United States

## Abstract

In 2021, Americans age 80 and older reported median fraud losses of $1,300 per incident, substantially higher than younger age groups (Federal Trade Commission, 2022). Using data from the Federal Trade Commission’s Sentinel Fraud Complaint Database and the Area Deprivation Index, this study assesses the demographic and contextual correlates of consumer fraud victimization in the United States. Models show a positive association between living in a disadvantaged community and reporting victimization (versus reporting no losses after being targeted). Black and Hispanic consumers are significantly more likely report victimization, although non-Hispanic White victims report higher average fraud losses. Results also show that older adults are significantly less likely to report fraud victimization relative to people age 30 and younger, but older victims lose hundreds of dollars more, on average. Applying a sentiment analysis to the consumer complaint narratives, we find that compared to emotionally neutral complaints, emotionally positive and emotionally negative complaints are positively associated with reporting victimization versus reporting no losses. Gift cards are the most common method of payment demanded by fraud criminals overall, but victims who pay using cash, wire transfer, or cryptocurrency lose significantly more money, on average. To limit the impact of fraud on the aging population, consumer protection agencies need to focus their efforts on educating older adults and members of socially and economically disadvantaged communities about scams, as well as enforce greater controls on cryptocurrency exchanges and retail gift card sales.

